# Prenylated Xanthones from the Bark of *Garcinia xanthochymus* and Their 1,1-Diphenyl-2-picrylhydrazyl (DPPH) Radical Scavenging Activities 

**DOI:** 10.3390/molecules15107438

**Published:** 2010-10-22

**Authors:** Yu Chen, Hua Fan, Guang-zhong Yang, Yan Jiang, Fang-fang Zhong, Hong-wu He

**Affiliations:** 1 Key Laboratory of Pesticide and Chemical Biology, Ministry of Education, Central China Normal University, Wuhan 430079, China; 2 State Key Laboratory of Drug Research, Shanghai Institute of Materia Medica, Chinese Academy of Sciences, Shanghai 201203, China; 3 College of Chemistry and Material Sciences, South Central University for Nationalities, Wuhan 430074, China; 4 Laboratory for Natural Product Chemistry, College of Pharmacy, South Central University for Nationalities, Wuhan 430074, China

**Keywords:** Xanthones, *Garcinia xanthochymus*, DPPH radical scavenging activity

## Abstract

*Garcinia xanthochymus* has been widely used in traditional Chinese medicine for expelling worms and removing food toxins. Bioassay-guided fractionation of an EtOAc-soluble extract of *G. xanthochymus* stem bark led to the isolation of six new xanthones. Their structures were elucidated by spectroscopic methods, especially 2D-NMR techniques. Free-radical-scavenging activities of the isolated compounds were elucidated through DPPH method. Most of the isolated compounds showed considerable free radical scavenging activity on DPPH assay. Compound **1** exhibited effective antioxidant scavenging activity against DPPH radical with an IC_50_ value of 19.64 μM, and compound **6** showed the lowest activity among all the tested molecules, with an IC_50_ value of 66.88 μM. These findings support the notion that the plant genus *Garcinia* is a good source of bioactive compounds.

## 1. Introduction

Reactive oxygen species (ROS) such as superoxide anion radical (O^-^·_2_) and hydroxyl radical (·OH) play an important role in the human body, and they are linked to the pathology of cirrhosis, cancer, and neurodegenerative diseases [1,2]. Oxidation could damage DNA, proteins, lipids and other small molecules. In order to prevent oxidative reactions in biological tissues against molecular targets, various synthetic or natural antioxidants can be used. However, synthetic antioxidants are not used extensively due to their toxicity and unwanted side effects. It has been suggested that natural antioxidants are safer and healthier than synthetic antioxidants. Therefore, more and more attention has been paid to the use of naturally occurring antioxidants for treatment or prophylaxis of various oxidative stress-related diseases [3]. Phenolic compounds play an important role in the antioxidative properties of many plant-derived antioxidants and they were also reported to possess a wide range of biological effects, such as antioxidant, antimicrobial, anti-inflammatory and vasodilatory actions [4]. 

**Figure 1:**
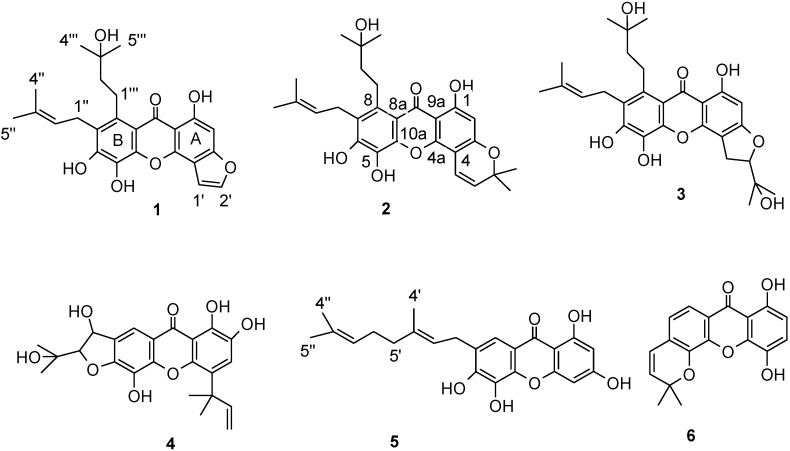
Structures of compounds **1**-**6**.

The genus *Garcinia* belongs to the Guttiferae family, which comprises 200 species confined to the tropics as trees or shrubs, and rarely subshrubs. It is well known to be a rich source of oxygenated and prenylated xanthones [5]. Xanthones are a class of polyphenolics that exhibit well-documented pharmacological properties, such as antioxidative, antileukaemic, antitumour, antiulcer, antimicrobial, antihepatotoxic, and CNS depressant activities [6], mainly due to their oxygenated heterocyclic nature and diversity of functional groups [7]. *Garcinia xanthochymus* is a traditional *Dai* medicine native to the south and southwest of Yunnan Province, P. R. China which can grow up to 10-20 m. It has been widely used as a traditional medicine for expelling worms and removing food toxins [8]. Previous phytochemical studies of the leaves, seeds, fruits, twig bark, and wood have demonstrated the presence of benzophenones [9,10,11,12,13,14,15], flavonoids [16,17], triterpenes [18] and xanthones [19,20,21]. In order to clarify the bioactive components, bioassay-guided fractionation has led to the isolation of six novel xanthones **1**-**6** (Figure 1). Herein we report the isolation and structural elucidation of these new xanthones and DPPH-radical scavenging activities of the isolated compounds.

## 2. Results and Discussion

### 2.1. Structural elucidations of xanthones

**Figure 2:**
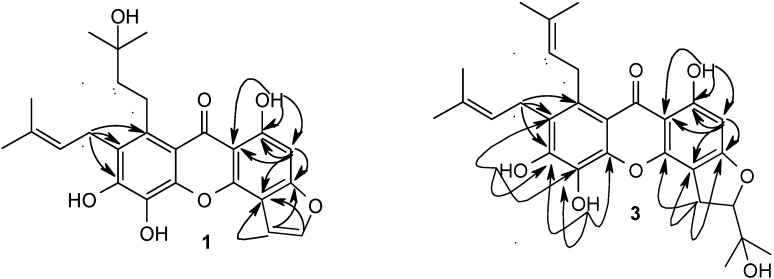
Significant HMBC correlations of compound **1** and **3**.

Compound **1** was obtained as a yellow powder. Its molecular formula C_25_H_26_O_7_ was determined by the molecular ion peak at *m/z* 438.1698 in the HREIMS (calcd 438.1679). The UV spectrum of **1** had characteristic xanthone absorptions at 231, 265, 350 nm. In the ^1^H-NMR spectrum of **1**, three hydroxyl groups [δ_H_ 8.90, 9.12(1H each, s) and 13.61(1H, s, chelated)] and an aromatic proton [δ_H_ 6.85 (1H, s)] appeared, in addition to a 3-methyl-2-butenyl group, a 3-hydroxy-3-methylbutyl group and a fused furan ring [δ_H_ 7.39, 7.80 (1H each, br s)]. The presence of the fused furan ring was substantiated by the methine carbons (δ_c_ 104.9 and 144.8) in the ^13^C-NMR spectrum. The HMBC correlations of the hydrogen-bonded proton (1-OH) with an oxygenated aromatic carbon at δ_c_ 160.9, a quaternary aromatic carbon at δ_c_ 105.8 and a methine aromatic carbon at δ_c_ 94.1 corresponding to an aromatic proton [δ_H_ 6.85 (1H, s)] in HSQC spectrum. It suggested that this proton may be attributed to H-2. The position of the furan ring was determined as follow. In the HMBC spectrum, one proton signal at δ_H_ 7.39 (1H , br s) of the furan ring showed correlations with a quaternary aromatic carbon at δ_c_ 108.5 (C-4) and an oxygenated aromatic carbon at δ_c_ 160.6 (C-3). The signals at δ_c_ 108.5 and 160.6 also correlated with the aromatic proton signal at δ_H_ 6.85 (1H, s, H-2). Therefore, the furan ring was fused at C-4 through an oxygen at C-3. The locations of other substituents were determined as follows. In the ^13^C-NMR spectrum, the aromatic carbons with an oxygen function were observed at δ_c_ 149.4, 130.3 and 150.5, which suggested the presence of a 1, 2, 3-trioxygenated benzene ring in partial structure B. In HMBC spectrum (Figure 2), the correlations of H_2_-1′′/C-6 (δ_C_ 150.5), C-7 (δ_C_ 126.1) and C-8 (δ_C_ 136.4) indicated that one 3-methyl-2-butenyl group was located at C-7. Thus, the remaining 3-hydroxy-3-methylbutyl group should be located at C-8. Compound **1** was thus identified to be 1,5,6-trihydroxy-7-(3-methyl-2-butenyl)-8-(3-hydroxy-3-methylbutyl)furano(2′,3′:3,4) xanthone.

Compound **2** was obtained as a yellow powder. The molecular formula was determined as C_28_H_32_O_7_ (*m/z* 480.2118) by HREIMS. Comparing its ^13^C-NMR and DEPT data with those of **1**, it was found that compound **2** had almost the same chemical shifts as those of **1**, except for the dimethylpyran ring carbon signals at δ_c_ 115.9 (d), 127.2 (d), 78.3 (s), 28.0 (q) and 28.0 (q) in **2** instead of furan ring carbon signals at δ_c_ 104.9 (d) and 144.8 (d) in **1**. These facts suggested that dimethylpyran ring in the structure of **2** replaced furan ring found in **1**. Thus, **2** was identified to 1,5,6-trihydroxy-7-(3-methyl-2-butenyl)-8-(3-hydroxy-3-methylbutyl)–6′, 6′-dimethylpyrano (2′,3′:3,4) xanthone.

Compound **3** was obtained as a yellow powder, whose molecular formula was determined as C_28_H_34_O_8_ by the HREIMS (*m/z* 498.2256, calcd. 498.2254). Comparison of NMR data of **3** with those of **1** indicated that the two compounds were closely related. The obvious spectroscopic differences between them resulted from the presence of a 2-(1-hydroxy-1-methylethyl)-2,3-dihydrofuran ring in **3**, instead of furan ring in **1**. The location of 2-(1-hydroxy-1-methylethyl)-2,3-dihydrofuran ring was fused at C-3 and C-4 of the xanthone nucleus with an ether linkage at C-3 by the HMBC correlations (Figure 2) between signals at δ_H_ 6.19 (H-2) to δ_C_ 163.7(C-1), 166.7(C-3) and δ_H_ 3.27(H_2_-1') to δ_C_ 166.7 (C-3), 102.5 (C-4). Therefore, the structure of **3** was determined as 1,5,6-trihydroxy-7-(3-methyl-2-butenyl)-8-(3-hydroxy-3-methylbutyl)–5′-(1-hydroxy-1-methylethyl)-4′, 5′-dihydrofurano (2′,3′:3,4) xanthone.

Compound **4** was obtained as a yellow amorphous powder. The [M^+^] at *m/z* 428.1464 in the HREIMS corresponds to C_23_H_24_O_8_ (calcd 428.1472). The ^1^H-NMR spectrum of **4** exhibited one chelated hydroxy group [δ_H_ 13.0 (1H, s)], two aromatic proton as a singlet at δ_H_ 7.34 (1H, s) and δ_H_ 7.79 (1H, s), a typical signal of a 1,1-dimethylallyl group at δ_H_ 6.36 (1H, dd, *J* = 17.7,10.8 Hz); 5.16 (1H, d, *J* = 17.7 Hz); 5.02 (1H, d, *J* = 10.8 Hz) and 1.65 (6H, s), as well as a 2-(1-hydroxy-1-methylethyl)-2, 3-dihydrofuran-3-ol moiety from resonances at δ_H_ 5.53 (1H, d, *J* = 3.9 Hz); 4.49 (1H, d, *J* = 3.9 Hz); 1.32 (3H, s) and 1.30 (3H, s). The ^13^C-NMR and DEPT experiments displayed the presence of four methyl, five methine, one methylene, 12 quaternary carbons and one carbonyl. A partial structure A of **4**, 1, 2-dihydroxy-4-(1, 1-dimethylallyl)xanthone, was deduced by comparison of the ^1^H-NMR and ^13^C-NMR data of **4** with those of subelliptenone H [22], and supported by a HMBC experiment (Table 3). The position of 2, 3-dihydrofuran ring was determined as follow. In HMBC spectrum, a singlet aromatic proton at δ_H_ 7.79 caused cross-peaks with δ_C_183.0 (C-9), which suggested that this proton was assigned to C-8. The aromatic carbons with an oxygen function were observed at δ_c_ 146.6, 129.6 and 154.0 in ^13^C NMR spectrum, which indicated the presence of a 1, 2, 3-trioxygenated benzene ring in partial structure B. Therefore, the 2, 3-dihydrofuran ring was fuse at C-7 through an oxygen at C-6. Thus, the structure of **4** was determined as 1, 2, 5, 4′-tetrahydroxy-4-(1,1-dimethylallyl)-5′-(2-hydroxypropan-2-yl)-4′, 5′-dihydrofurano-(2′, 3′ : 6, 7)xanthone.

Compound **5** was obtained as a yellow powder, whose molecular formula was determined as C_23_H_24_O_6_ by the HREIMS (*m/z* 396.1574, calcd. 396.1573). By comparing the ^1^H-NMR spectrum of **5** with that of the previously isolated compounds from the same plant, **5** was identified to be an isomer of 1, 2, 5, 6-tetrahydroxy-7-geranylxanthone [21]. The geranyl group was located at C-7 based on the HMBC correlations (Table 3) between δ_H_ 7.51 (H-8) with δ_C_ 180.7(C-9), 150.4(C-6), 29.1(C-1') and δ_H_ 3.43 (H_2_-1') with δ_C_126.5 (C-7). The ^1^H NMR spectrum of **7** exhibited one chelated hydroxy group [δ_H_ 13.22 (1H, s)], two *meta*-aromatic proton at δ_H_ 6.20 (1H, br s) and δ_H_ 6.40 (1H, br s). Therefore, a coupling of *meta*-aromatic protons were assigned to C-2 and C-4 respectively, which was further supported by the HMBC correlations of H-2 with δ_C_ 165.5(C-3), 94.3(C-4) and 102.8(C-9a). Based on the above observation, the structure of **5** was established as 1, 3, 5, 6-tetrahydroxy-7-geranylxanthone.

**Table 1:** ^1^H-NMR data of compounds **1**-**3** and **6**.

Position	1	2	3	6
1-OH	13.61 s		14.03 s	12.02 s
5-OH	8.90 s		9.43 s	
6-OH	9.12 s		9.82 s	
2	6.85 s	6.14 s	6.19 s	6.64 d (8.9)
3				7.32 d (8.9)
7				7.18 d (8.1)
8				7.72 d (8.1)
1′	7.39 br s	7.11 d (9.3)	3.27 m	6.59 d (9.8)
2′	7.80 br s	5.72 d (9.3)	4.77 m	6.02 d (9.8)
4′		1.42 s	1.17 s	1.54 s
5′		1.42 s	1.17 s	1.54 s
1′′	3.56 d (5.6)	3.35 d (6.0)	3.39 d (5.4)	
2′′	5.14 br s	5.15 br s	5.02 br s	
4′′	1.83 s	1.84 s	1.77 s	
5′′	1.68 s	1.70 s	1.65 s	
1′′′	3.45 m	3.44 m	3.34 m	
2′′′	1.76 m	1.74 m	1.55 m	
4′′′	1.32 s	1.32 s	1.20 s	
5′′′	1.32 s	1.32 s	1.20 s	

Compound **6** was obtained as a yellow powder. The molecular formula was determined as C_18_H_14_O_5_ (*m/z* 310.0848) by HREIMS. The ^13^C NMR spectrum showed 18 carbon signals, which were classified into 2 methyl, 6 methine and 10 quaternary carbons by analysis of the DEPT spectra. The ^1^H NMR data showed two sets of *ortho*-aromatic protons at δ_H_ 6.64 (1H, d, *J* = 8.9 Hz) and 7.32 (1H, d, *J* = 8.9 Hz); 7.18 (1H, d, *J* = 8.1 Hz) and 7.72 (1H, d, *J* = 8.1 Hz), one chelated hydroxyl group at δ_H_ 12.02 (1H, s), one *cis* olefinic group [δ_H_ 6.59 (1H, d, *J* = 9.8 Hz), 6.02 (1H, d, *J* = 9.8 Hz)] as well as two tertiary methyls attached to an oxygenated carbon [δ_H_ 1.54 (6 H, s)] indicating the presence of a dimethylpyran ring system. The positions of the substituents were deduced by analysis of HMBC. In the HMBC spectrum, there were correlations between the chelated hydroxyl group at δ_H_ 12.02 and the carbon signal at δ_c_ 154.2 (C-1) and 109.6 (C-2) corresponding to one of the *ortho*-coupled proton δ_H_ 6.64 (1H, d, *J* = 8.9 Hz) in the HSQC spectrum. These results indicated that **6** was a 1, 4-dihydroxyxanthone derivative. The other coupling of *ortho*-aromatic protons at δ_H_ 7.18 (1H, d, *J* = 8.1 Hz) and 7.72 (1H, d, *J* = 8.1 Hz) were assigned as H-7 and H-8, respectively, by the HMBC correlation of δ_H_ 7.72 with carbonyl carbon C-9 (182.4). The dimethylpyran ring was fused with the xanthone in an angular form which was further supported by HMBC correlation of δ_H_ 6.59 (1H, d, *J* = 9.8 Hz) with δ_c_ 141.7 (s, C-5), 127.6 (s, C-6) and 122.3(d, C-7) and δ_H_ 6.02 (1H, d, *J* = 9.8 Hz) with δ_c_ 127.6 (s, C-6). Thus, the structure of compound **6** was established as 1, 4-dihydroxy-6′, 6′-dimethylpyrano (2′, 3′: 5, 6) xanthone.

**Table 2:** ^13^C-NMR data of compounds **1-3** and **6**.

Position	1	2	3	6
1	160.9 (qC)	163.8 (qC)	163.7 (qC)	154.2 (qC)
2	94.1 (CH)	99.0 (CH)	92.4 (CH)	109.6 (CH)
3	160.6 (qC)	160.3 (qC)	166.7 (qC)	124.5 (CH)
4	108.5 (qC)	104.0 (qC)	102.5 (qC)	138.2 (qC)
4a	146.1 (qC)	150.9 (qC)	150.4 (qC)	144.6 (qC)
5	130.3 (qC)	130.0 (qC)	129.7 (qC)	141.7 (qC)
10a	149.4 (qC)	146.8 (qC)	145.8 (qC)	146.1 (qC)
6	150.5 (qC)	151.3 (qC)	150.6 (qC)	127.6 (qC)
7	126.1 (qC)	125.6 (qC)	124.8 (qC)	122.3 (CH)
8	136.4 (qC)	136.7 (qC)	134.9 (qC)	117.3 (CH)
8a	112.0 (qC)	111.6 (qC)	110.1 (qC)	121.5 (qC)
9	183.8 (qC)	183.1 (qC)	181.9 (qC)	182.4 (qC)
9a	105.8 (qC)	101.1 (qC)	103.3 (qC)	109.4 (qC)
1′	104.9 (CH)	115.9 (CH)	26.7 (CH_2_)	122.1 (CH)
2′	144.8 (CH)	127.2 (CH)	91.6 (CH)	134.9 (CH)
3′		78.3 (qC)	70.0 (qC)	78.6 (qC)
4′		28.0 (CH_3_)	26.0 (CH_3_)	27.6 (CH_3_)
5′		28.0 (CH_3_)	24.9 (CH_3_)	17.6 (CH_3_)
1′′	25.2 (CH_2_)	25.1 (CH_2_)	24.1 (CH_2_)	
2′′	123.7 (CH)	123.7 (CH)	123.3 (CH)	
3′′	131.5 (qC)	131.4 (qC)	130.6 (qC)	
4′′	18.0 (CH_3_)	17.9 (CH_3_)	18.0 (CH_3_)	
5′′	25.6 (CH_3_)	25.6 (CH_3_)	25.6 (CH_3_)	
1′′′	24.8 (CH_2_)	24.8 (CH_2_)	24.5 (CH_2_)	
2′′′	45.3 (CH_2_)	45.3 (CH_2_)	44.9 (CH_2_)	
3′′′	70.0 (qC)	69.9 (qC)	69.0 (qC)	
4′′′	28.9 (CH_3_)	28.9 (CH_3_)	29.1 (CH_3_)	
5′′′	28.9 (CH_3_)	28.9 (CH_3_)	29.1 (CH_3_)	

**Table 3:** ^1^H- and ^13^C-NMR, HMBC data of compounds **4**-**5** in acetone-d_6__._

	4				5		
	δ_H_	δ_C_	HMBC		δ_H_	δ_C_	HMBC
1		148.0				164.4	
2		139.6			6.20 br s	98.4	C-3, 9a, 4
3	7.34 s	122.4	C-2, 4a, 1′			165.5	
4		126.1			6.40 br s	94.3	
4a		147.4				158.3	
5		129.6				132.1	
10a		146.6				145.3	
6		154.0				150.4	
7		126.1				126.5	
8	7.79 s	112.8	C-9		7.51 s	116.1	C-9, 6, 10a, 1′
8a		115.2				113.4	
9		183.0				180.7	
9a		109.0				102.8	
1′		40.5			3.43 d (6.6)	29.1	C-7, 2′, 3′
2′	6.36dd (17.7,10.8)	147.8			5.41 t (6.6)	122.4	C-7, 1′, 4′, 5′
3′	5.16 d (17.7)	110.9	C-1′			136.8	
	5.02 d (10.8)						
4′	1.65 s	27.3	C-2′, 3′, 4		1.74 s	15.9	C-2′, 3′, 5′
5′	1.65 s	27.2	C-2′, 3′, 4		2.08 m	40.2	C-2′, 3′, 2′′
1′′	5.53 d (3.9)	72.4			2.12 m	28.3	C-2′′, 3′′
2′′	4.49 d (3.9)	99.8			5.13 t (6.0)	124.7	C-5′, 1′′, 4′′, 5′′
3′′		70.9				131.5	
4′′	1.32 s	25.2	C-2′′, 3′′		1.63 s	25.5	C-2′′, 3′′, 5′′
5′′	1.30 s	25.5	C-2′′, 3′′		1.58 s	17.4	C-2′′, 3′′, 5′′
1-OH	13.0 s		C-2		13.22 s		

### 2.2. DPPH radical-scavenging activities of the purified compounds

The six xanthones were evaluated for their antioxidant activities by DPPH free radical scavenging method (Table 4). Most of the isolated compounds showed considerable free radical scavenging activity on DPPH assay. The potency of DPPH radical-scavenging activity was in a decreasing order: **1** > **3** > **2** > **5**> **4** > **6**. 

**Table 4:** *In vitro* DPPH radical scavenging activities of prenylated xanthones isolated from the bark of *G. xanthochymus.*

Compound	DPPH radical-scavenging activity (IC_50_. μM )
**1**	19.64 ± 0.39
**2**	31.82 ± 0.08
**3**	22.07 ± 0.25
**4**	40.70 ± 0.10
**5**	34.27 ± 0.25
**6**	66.88 ± 0.19
ascorbic acid	13.16 ± 0.03
gallic acid	5.86 ± 0.03

Compound **1** exhibited effective antioxidant scavenging activity against DPPH radical, with an IC_50_ value of 19.64 μM, and compound **6** showed the lowest activity with an IC_50_ value of 66.88μM among all the tested molecules. The DPPH radical scavenging activities of these compounds seemed to be related to the number of phenol-like OH groups at the xanthone skeleton. It was reported previously that the radical scavenging activity was increased in the presence of an increasing number of phenol like OH groups in a molecule [23]. However, compound **5**, having four phenol-like OH groups, showed a lower radical scavenging activity compared to that of compound **1** having three phenol-like OH groups. This was because the presence of furan ring in compound **1** extended the conjugation system to participate in stabilizing the phenoxy radical by resonances, therefore increasing the radical-scavenging activity of compound **1** [24]. From above the data, it can be deduced that the main components responsible for the antioxidant activities of *Garcinia xanthochymus* were the phenolic compounds, such as xanthone derivatives.

## 3. Experimental

### 3.1. General

UV spectra were measured on an SP-2102UVPC spectrometer using MeOH as the solvent. NMR spectra were run in DMSO-*d_6_* or Me_2_CO-*d_6_* on a Bruker AM-400 (1D) or Varian Inova-600 (2D) spectrometer with TMS as an internal standard. EIMS and HREIMS measurements were conducted with a Finnigan MAT 95 instrument. Thin-layer chromatography (TLC) was performed on silica gel 60 GF_254_, while column chromatography was carried out using silica gel (200-300 mesh) from Qingdao Haiyang Chemical Group Co., P. R. China and C_18_ reversed-phase silica gel from YMC CO., LTD., Japan. 

### 3.2. Plant material

The bark of *Garcinia xanthochymus* was collected from Xishuangbanna Prefecture, Yunnan Province, P.R. China and identified by Xishuangbanna Prefecture National Medicine Research Institute. The voucher specimen (06061201) was deposited with the Herbarium of College of Pharmacy, South Central University for Nationalities.

### 3.3. Extraction and isolation procedures

The powdered bark of *G. xanthochymus* (6.5 kg) was extracted with 95% EtOH (25 L × 3) and then successively partitioned with petroleum ether (P.E.) (3.0 L × 3), EtOAc (3.0 L × 3) and *n*-BuOH (3.0 L × 3). The combined EtOAc extract (590 g) was chromatographed on silica gel with P.E-Me_2_CO (9:1, 8:2, 7:3, 1:1, 3:7, 0:1, *v/v*) to give thirteen fractions (fr.1−fr.13). Fr.6 (17.0 g) was separated on a silica column (toluene/Me_2_CO 95:5→3:7 gradient system), and then purified by chromatography on a silica gel (CHCl_3_-MeOH, 1:0→1:1 gradient system) and RP-18 (MeOH-H_2_O, 8:2) to afford compound **6** (2.8 mg). Fr.7 (33.8 g) was extensively separated over a silica column (toluene/Me_2_CO 95:5→3:7 gradient system) and RP-18 (MeOH-H_2_O, 3:7→7:3 gradient system) to afford **1** (3.2 mg), **2** (9.8 mg) and **5** (4.8 mg). Fr. 9 (10.8 g) was also subjected to silica gel with a gradient elution (toluene-Me_2_CO, 9:1→3:7 gradient system) and RP-18 (MeOH-H_2_O, 3:7→7:3 gradient system) to afford compounds **3** (7.4 mg) and **4** (8.8 mg). 

### 3.4. Physical data of new compounds

*1,5,6-Trihydroxy-7-(3-methyl-2-butenyl)-8-(3-hydroxy-3-methylbutyl)–furano(2′,3′:3,4) xanthone* (**1**). Yellow amorphous powder; UV λ_max_ (MeOH) nm (logε): 231 (3.50), 265 (3.49), 350 (3.58); For ^1^H-NMR and ^13^C-NMR spectroscopic data (in Me_2_CO-*d_6_*), see Table 1 and Table 2; EIMS (70 eV) *m/z* (%): 438 (M^+^, 28), 420 (36), 377 (76), 364 (48), 349 (100), 323 (80); HREIMS *m/z* 438.1698 (calcd. for C_25_H_26_O_7_, 438.1679).

*1,5,6-Trihydroxy-7-(3-methyl-2-butenyl)-8-(3-hydroxy-3-methylbutyl)–6′,6′-dimethylpyrano (2′,3′ 3,4) xanthone* (**2**). Yellow amorphous powder; UV λ_max_ (MeOH) nm (logε): 229 (3.52), 263 (3.52), 350 (3.63); For ^1^H-NMR and ^13^C-NMR spectroscopic data (in Me_2_CO-*d_6_*), see Table 1 and Table 2; EIMS (70 eV) *m/z* (%): 480 (M^+^, 36), 463 (76), 447 (100), 419 (72), 391(84), 365 (64), 349 (56); HREIMS *m/z* 480.2118 (calcd. for C_28_H_32_O_7_, 480.2148).

*1,5,6-Trihydroxy-7-(3-methyl-2-butenyl)-8-(3-hydroxy-3-methylbutyl)–5′-(1-hydroxy-1-methyl-ethyl)-4′,5′-dihydrofurano(2′,3′:3,4) xanthone* (**3**). Yellow amorphous powder; UV λ_max_ (MeOH) nm (logε): 250 (4.21), 285 (4.00), 334 (4.11); For ^1^H-NMR and ^13^C-NMR spectroscopic data (in DMSO-*d_6_*), see Table 1 and Table 2; EIMS (70 eV) *m/z* (%): 498 (M^+^, 8), 480 (40), 437 (100), 424 (44), 409 (52), 383 (100), 365 (32); HREIMS *m/z* 498.2256 (calcd. for C_28_H_34_O_8_, 498.2254).

*1,2,5,4′-Tetrahydroxy-4-(1,1-dimethylallyl)–5′-(2-hydroxypropan-2-yl)-4′,5′-dihydro furano-(2′,3′:6,7) xanthone* (**4**). Yellow amorphous powder; UV λ_max_ (MeOH) nm (logε): 230 (3.46), 264 (3.47), 337 (3.46), 389(sh)(3.09); For ^1^H-NMR and ^13^C-NMR spectroscopic data (in Me_2_CO-*d_6_*), see Table 3; EIMS (70 eV) *m/z* (%): 428 (M^+^, 8), 410 (6), 392 (16), 352 (56), 319 (56), 319 (100); HREIMS *m/z* 428.1464 (calcd. for C_23_H_24_O_8_, 428.1472).

*1,3,5,6-Tetrahydroxy-7-geranylxanthone* (**5**). Yellow amorphous powder; UV λ_max_ (MeOH) nm (logε): 260 (4.00), 341(4.01); For ^1^H-NMR and ^13^C-NMR spectroscopic data (in Me_2_CO-*d_6_*), see Table 3; EIMS (70 eV) *m/z* (%): 396 (M^+^, 28), 327 (32), 311 (100), 274 (56), 123 (181), 69 (24); HREIMS *m/z* 396.1574 (calcd. for C_23_H_24_O_6_, 396.1573).

*1,4-Dihydroxy-6′,6′-dimethylpyrano (2′,3′:5,6) xanthone* (**6**). Yellow amorphous powder; UV λ_max_ (MeOH) nm (logε): 230 (3.33), 264 (3.33), 354 (3.45), 400 (sh) (3.26); For ^1^H-NMR and ^13^C-NMR spectroscopic data (in Me_2_CO-*d_6_*), see Table 1 and Table 2; EIMS (70 eV) *m/z* (%): 310 (M^+^, 36), 295 (100), 148 (15); HREIMS *m/z* 310.0848 (calcd. for C_18_H_14_O_5_, 310.0841).

### 3.5. DPPH radical scavenging activity

Scavenging activities of the purified compounds from *G. xanthochymus* towards DPPH radical were assessed by using the method described by Scherer and Godoy with a slight modification [25,26]. Briefly, a 0.08 mM solution of DPPH radical solution in methanol was prepared and then, the solvent extracts and purified compounds at different concentrations (0.1 mL) were added to the prepared DPPH radical solution (3.9 mL); the mixture was shaken vigorously, after a 30 min incubation period at 37 ºC in the dark, the absorbance was measured at 517 nm by using a UV-visible spectrophotometer. Obviously, decreasing of the DPPH solution absorbance indicates an increase of the DPPH radical-scavenging activity. The radical scavenging activity is given as DPPH radical scavenging effect that is calculated using equation (1):

DPPH radical scavenging effect (%) = [(A_0_-A_1_)/A_0_] × 100 (1)

where A_0_ was the absorbance of control and A_1_ was the absorbance in the presence of the standard, solvent extracts or purified compounds at different concentrations. Ascorbic acid (V_C_) and gallic acid were used as positive controls, respectively. All the tests were performed in triplicate. The scavenging activities of the purified compounds towards DPPH radical were expressed as IC_50_, which was determined to be the effective concentration at which DPPH radical was scavenged by 50%. The IC_50_ value was obtained by interpolation from linear regression analysis.

### 3.6. Statistical analyses of results of activity studies

The results were performed as mean ± standard deviation (SD) of three determinations. Analysis of significance differences among means were tested by one-way analysis of variance. The IC_50_ values were calculated by linear regression analysis. 

## 4. Conclusions

In the course of our ongoing research project on bioactive natural products from *G. xanthochymus*, an EtOAc-soluble partition of the EtOH extract of the bark of *G. xanthochymus* displayed significant antioxidant activity in the 1,1-diphenyl-2-picrylhydrazyl (DPPH) scavenging bioassay. This prompted us to perform a detailed bioassay-guided isolation from this plant, which led to the isolation of six new xanthones. Notably, most of the isolated compounds showed considerable free radical scavenging activity in the DPPH assay. Compound **1** exhibited effective antioxidant scavenging activity against DPPH radicals with an IC_50_ value of 19.64 μM, and compound **6** showed the lowest activity among all the tested molecules, with an IC_50_ value of 66.88 μM. These findings support that plant genus *Garcinia* is a good source of bioactive compounds.
